# Phase 0 - Microdosing strategy in clinical trials

**DOI:** 10.4103/0253-7613.45147

**Published:** 2008

**Authors:** P. Usha Rani, M. U. R. Naidu

**Affiliations:** Department of Clinical Pharmacology and Therapeutics, Nizam's Institute of Medical Sciences, Hyderabad, India

**Keywords:** Drug discovery and development, microdosing, phase 0

## Abstract

Drug development is an activity that is long, complex and expensive. In 2004, attrition in the drug development paradigm prompted the US Food and Drug Administration (FDA) to introduce its ‘Critical Path’ document, which highlighted the serious discordance between major scientific advances and limited drug development process. One issue addressed was that of microdosing. The concept of microdosing involves the use of extremely low, nonpharmacologically active doses of a drug to define the pharmacokinetic profile of the medication in human subjects. Microdosing, thus, appears as a new viable concept in the ‘toolbox’ of the drug development activity. It appears that microdosing strategy could complement standard animal-to-human scaling, redefining the existing concept of phase I clinical research. In future, when research methods and technology involved in Phase 0 studies become more sophisticated, human microdosing may be applied to a number of drugs developed subsequently.

## Introduction

Human drug development is a dynamic process that keeps pace with the recent advances within the pharmaceutical analytical labs, combined with a precise understanding of the integrated pharmacokinetic-pharmacodynamic-pharmacogenomic drug profile. New drugs are a great need for many clinical conditions but, unfortunately, development costs are rising and the number of drugs receiving marketing approval has fallen. Further, drug development is a long, complex and expensive activity. It typically involves a total cost of US$ 500 million to 1 billion per marketed drug and is spread over 10-15 years.[1] In the past decade, attrition was the highest (62%) during Phase II, 45% during phase III and significant (23%) at the time of registration.[2] The situation has become so serious that the FDA published the document ‘critical path’, highlighting the problems in drug development and encouraging novel approaches to be incorporated into the current drug development paradigm.[3–5]

One important attribute of a drug is its pharmacokinetic profile. Suboptimal pharmacokinetics is stated to be a reason for failures during drug development. Too low concentrations of drug at the target organ for lesser time can lead to efficacy failures, while wrong concentrations reaching wrong targets for longer time may lead to toxicity. Thus, a new experimental approach has been developed, known as Phase 0 or microdosing studies, to address issues pertaining to drug metabolism and pharmacokinetics.

Microdosing methodology is referred to as a new viable ‘tool’ in the drug development ‘toolbox’. In microdosing, extremely low, nonpharmacologically active doses of a drug are used to define the agent's pharmacokinetic profile in humans.[6] Thus, by definition, microdosing means use of ‘less than 1/100^th^ of the dose calculated to yield a pharmacological effect of the test substance to a maximum dose of < 100 micrograms (European Medicines Agency paper).’ However, in addition to this, the US FDA suggests a maximum microdose of < 30 nanomoles for protein products.[7,8] Microdosing, therefore, allows not only for the selection of the drug candidates more likely to be developed successfully, but also for the determination of the first dose for the subsequent Phase I clinical trial.

## Microdosing and Technical Advances

A new approach using big physics instrumentation to obtain human pharmacokinetic information before the usual expensive phase I safety program is conducted is the phase 0 microdosing.[6] It is hypothesized that microdosing will help to reduce or replace the extensive animal testing of compounds for kinetics, which may later be rejected in human studies. Thus, microdose studies use minute quantities of drug and are not intended to produce any pharmacologic effect, when administered to humans, and, therefore, may not cause any adverse events also, but may produce useful pharmacokinetic information and help in further development of the compound. However, ultrasensitive and specific analytical methods capable of measuring drug and metabolite concentrations in the low picogram to fentogram range are required. Most common of these are liquid chromatography coupled with tandem mass spectrometry (LC-MS-MS) and Accelerator Mass Spectrometry (AMS). The latter method is extremely sensitive compared to LC-MS-MS. However, compounds must be isotopically labeled, mostly with C^14^. This may be a disadvantage to the use of AMS, but a dose of radiolabeled compound use for human studies is as low as 100 nanocuries [Table 1].[9–11]

**Table 1:** Showing a comparison of microdosing strategy and conventional studies

*Features*	*Microdosing strategy*	*Conventional approach*
Time from preclinical to first in man studies	6-8 months	12-18 months
Cost of early phase of drug development	US$ 0.3 -.0.5 million	US$ 1.5-5.0 million
Amount of drug required	<100 micrograms	About 100 grams
Special requirements	C14 labeled compound, if using AMS	None required
Regulatory requirements	Very few and limited	Established firmly

## Microdosing and Regulatory Advances

The changes observed in the recent regulatory guidelines also stimulate the more frequent use of microdosing in subjects. Although regulatory authorities may not make microdosing a mandatory requirement as the data can be obtained by other methods, the regulatory agencies may provide approval or similar incentives for companies bringing certain drugs like life-saving medications to the development and commercialization stages more rapidly.

Further, accurate characterization of the kinetics of a drug over time, after administration, is an important regulatory requirement. This can be achieved by administration of radiolabeled drug to the subject and following its fate in plasma and excreta. Since AMS studies require very minute quantities of radiolabeled compounds, it may not be a significant source of material risk by regulatory authorities. Furthermore, recently, European Medicinal Agency and USA Federal Drugs Authority have published articles and supported evaluation.[4,5]

## Advantages of Microdosing

First of all, microdosing requires minute quantities of the drug for safety testing. A microdose is so small that when administered to human subjects, it is not intended to produce any pharmacologic action; hence, the risk of adverse events is less.

A smaller toxicology package is required. As per the regulatory requirement, animal studies, at least in one species, are required to establish microdose in humans, but at a much reduced level. Further, if human screening of compounds is done earlier in the drug development process, fewer animal studies are required before Phase I clinical trials. Thus, further animal studies can be avoided with compounds having unsuitable pharmacokinetic profiles.

From preliminary toxicology data of animals, pharmacokinetic data for the initial dose selection can be obtained in a short time of 4-6 months, whereas in Phase I studies it takes 12-18 months. During drug development, when a large number of me-too compounds are screened and found to have similar or differing animal pharmacokinetics, comparative human microdose studies can be done to establish pharmacokinetics. This pharmacokinetic data can further be used[1] to help in selection of the ideal candidate drug,[2] obtain the first in man dose of that selected drug[3] Establish the tentative pharmacological dose and[4] calculate the probable cost of the deliverable. A further question arises, which asks whether it is ethical to expose a subject to a pharmacological dose unnecessarily, when later, due to poor pharmacokinetics, the development of the drug will be halted.[5–7]

A rigorous assessment of the utility of microdosing studies to accurately predict drug disposition in small groups of humans should include a comparative trial pairing the pharmacokinetic results obtained from paired therapeutic dose and microdose data.[6] In a study performed to compare the pharmacokinetics of five drugs – warfarin, ZK253, diazepam, midazolam and erythromycin, administered at a microdose or pharmacological dose, the authors concluded that when used appropriately and intelligently, microdosing offers the potential to aid in early candidate selection.[12]

Further, the cost of conducting a microdose study is phenomenally less, as compared to a full Phase I study. A conventional phase I study may cost about US$ 1.5 to 3.0 million, whereas in the microdosing approach, the cost drops to about US$ 0.3 - 0.5 million.[13]

Human microdosing promises to be a significant analytical tool. In future, as research methods and technology involved in Phase 0 trials become more sophisticated, human microdosing may be applied to a number of drugs that could potentially be administered consequently.[13,14]

Additionally, microdosing could be useful in the discovery of endogenous biomarkers, which would assist in the quantitative evaluation of the *in vivo* effects of drugs.

Last but not the least, in oncology, phase 0 clinical trials are welcome in a big way. These studies are designed with the objective to establish at the very earliest opportunity – before a large numbers of patients have been accrued and exposed to potential drug-associated toxicity – whether an agent is modulating its target in a tumor, and consequently whether further clinical development is warranted.[14]

## Limitations of Microdosing

There are many questions which need to be answered regarding the predictive accuracy of microdosing. We still do not have enough studies to clearly exemplify whether the body's reaction to a particular compound is similar, when used as microdose and in its pharmacological dose; otherwise, it could lead to false negatives (compound being rejected) or false positives (compound acceptable based on microdose data but rejected subsequently when used in pharmacological doses).[15]

Another question of concern is whether microdosing predicts pharmacokinetic parameters accurately for drugs showing nonlinear kinetics. This aspect has been addressed in CREAM study (Consortium for Resourcing and Evaluating AMS Microdosing), where the disparity in warfarin disposition is described. Thus, caution should be exercised when microdosing is employed to drugs with complex pharmacokinetics, especially during early drug development of new chemical entities.[7–9]

Thus, a microdose may not be able to predict the behaviour of a clinical dose of the drug. However, though the CREAM study was not exhaustive, it demonstrated about 70% approximation between microdose and pharmacological dose pharmacokinetics.

Another limitation of microdosing relates to metabolism and stability of certain compounds. Some compounds dissolve readily at microdose, yielding good absorption characteristics; however, at therapeutic doses, they exhibit limited solubility, and absorption becomes dependent on the rate and extent of dissolution, which cannot be predicted at microdose levels.

Further, AMS (accelerator mass spectrometry) and PET (positron emission tomography) are applied to analyze the concentration of the drugs in low picogram to femotgram range, when microdose is used. These radiotracer assays have the disadvantages of short tracer half-life and limited specificity (as assay may include metabolites also). Both for PET and AMS, the drugs must be labeled at metabolically stable sites.[5,9,15]

The limitations of microdosing relate to compound metabolism and solubility of compound. Many processes within the body involve the use of specialized transporters, enzymes and binding sites, which can be saturated such that the pharmacokinetic profile is very different at the higher therapeutic dose than seen with the microdose.[16] Further, the compounds must be soluble to pass across the cell membranes and act within the body. Most compounds dissolve rapidly at microdose levels, yielding rapid and often extensive absorption. However, at higher therapeutic doses, many compounds exhibit limited solubility. This means, absorption becomes more dependent on the rate and extent of dissolution, which cannot be predicted by microdose. Thus, it has been suggested that the dose of 100 micrograms may be too low to achieve the full potential of microdosing.[17]

## Conclusion

Human microdosing clearly holds significant promise as an analytical tool. In the coming years, as research methods and technology involved in Phase 0 trials become more sophisticated, human microdosing may be applied to a number of drugs that could potentially be administered consecutively. Microdosing may later become an accepted approach in drug development, when first in man studies may begin with a Phase 0 study. However, the true utility of Phase 0 microdosing studies lies with the ability to predict under what circumstances this approach will provide data within a specified and acceptable range, as compared to the therapeutic dose data. Capitalizing on the continuing rapid advances in drug development technology, there is no question that decreasing the time of drug development, reduces the cost phenomenally. Until further information is available, we opine that microdosing strategy could complement the standard animal-to-human allometric scaling, redefining the present phase I study designs. This strategy may help to reduce animal testing in the identification of novel drug candidates.

Further, microdosing may help both patients and the pharma industry with earlier availability of new test medication and reduced attrition of compounds at later stages of drug development. Microdosing allows not only selection of drug candidates more likely to be developed successfully, but also helps in determination of the first dose for the subsequent Phase I clinical studies.
